# Progression to B acute lymphoblastic leukemia in 8p11 myeloproliferative syndrome with t(6;8)(q27;p12)

**DOI:** 10.1007/s12185-023-03577-z

**Published:** 2023-03-17

**Authors:** Fumi Nakamura, Sachiko Seo, Yasuhito Nannya, Rika Ayabe, Wataru Takahashi, Tomoyuki Handa, Honoka Arai, Hisako Iso, Yuko Nakamura, Yuka Nakamura, Ko Sasaki, Motoshi Ichikawa, Yoichi Imai, Seishi Ogawa, Kinuko Mitani

**Affiliations:** 1 Department of Hematology and Oncology, Dokkyo Medical University, Tochigi, Japan; 2 Department of Pathology and Tumor Biology, Kyoto University, Kyoto, Japan; 3 Division of Hematopoietic Disease Control, Institute of Medical Science, The University of Tokyo, Tokyo, Japan

**Keywords:** 8p11 syndrome, polycythemia vera, Acute B-lymphoblastic leukemia, *RUNX1*

## Abstract

8p11 myeloproliferative syndrome is a rare hematological malignancy caused by the translocation of FGFR1. Patients present with a myeloproliferative neoplasm that frequently transforms into acute myeloid leukemia or T-lymphoblastic lymphoma/leukemia. Here, we report a molecular study of a patient with 8p11 myeloproliferative syndrome who developed acute B-lymphoblastic leukemia and then transformed to mixed-phenotype acute leukemia. A 67-year-old woman was diagnosed with a myeloproliferative neoplasm with t(6;8)(q27;p12) and was monitored for polycythemia vera. Four years later, she developed acute B-lymphoblastic leukemia with an additional chromosomal abnormality of − 7. Despite two induction regimens, she failed to achieve complete remission, and leukemia transformed into mixed-phenotype leukemia. Targeted sequencing of serial bone marrow samples identified the RUNX1 L144R mutation upon transformation to B-cell leukemia. After those two induction regimens, some RUNX1 mutation-positive leukemic cells obtained the JAK2 V617F mutation, which was associated with the emergence of myeloid markers, including myeloperoxidase.

## Introduction

Eight p11 (8p11) myeloproliferative syndrome (EMS) is a rare hematological malignancy caused by the translocation of *FGFR1* located on chromosome 8p11-12. Thirteen partner genes of *FGFR1* have been reported [1]. The common fusion genes are *ZMYM2-FGFR1* and *BCR-FGFR1*. The generation of the fusion gene and the resultant expression of the fusion protein leads to constitutive activation of the FGFR1 tyrosine kinase. Patients with EMS present with a myeloproliferative neoplasm (MPN) associated with eosinophilia and lymphadenopathy and frequently progress to acute myeloid leukemia or T-lymphoblastic leukemia/lymphoma or rarely to acute B-lymphoblastic leukemia or mixed-phenotype acute leukemia [, , 2–4]. The median time for leukemic transformation is two months [2]. In general, transformed leukemia is refractory to chemotherapy, and thus, hematopoietic stem cell transplantation is the only curative treatment. The mechanism of transformation in EMS is largely unknown.

We present a case with 8p11 syndrome that progressed to acute B-lymphoblastic leukemia, which transformed into mixed phenotype leukemia after two induction regimens. We performed targeted sequencing of serial bone marrow samples to elucidate the molecular mechanisms underlying the development of acute leukemia in MPN.

## Case presentation

**Fig. 1:**
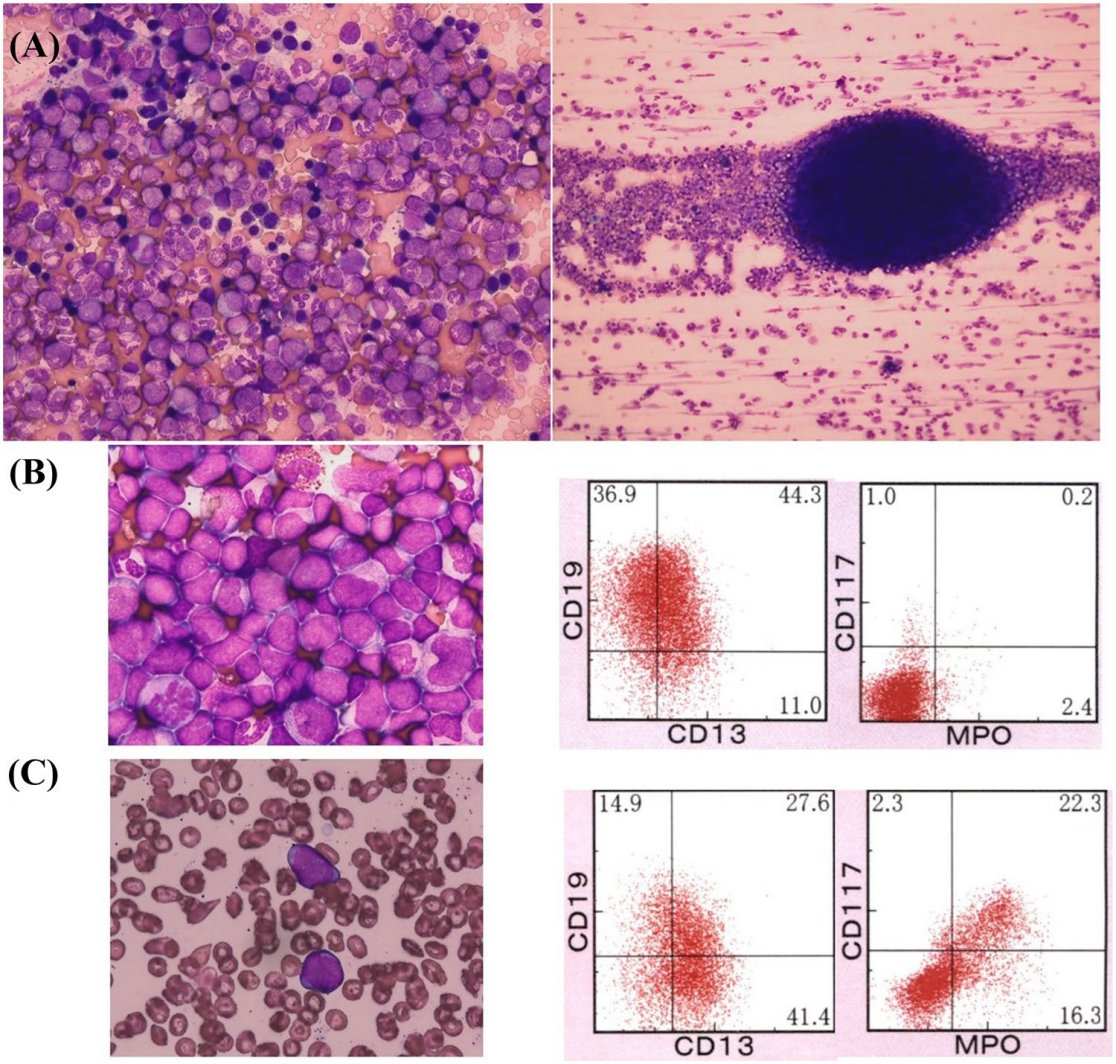
Images of bone marrow aspirate and flow cytometric analyses. **A** Low-power field (right) and high-power field (left) of the bone marrow aspirates at the time of polycythemia vera. **B** The bone marrow smear (left) and surface marker expression (right) at the diagnosis of B-ALL. **C** The bone marrow smear (left) and surface marker expression (right) at the transformation into mixed-phenotype

A 67-year-old woman was referred to Dokkyo Medical University Hospital for leukocytosis. Her white blood cell count was 35.3 × 10^9^/L (blast 0%, promyelocyte 0.5%, myelocyte 5.5%, metamyelocyte 6%, neutrophil 69%, eosinophil 3% and basophil 0%), red blood cell count was 6.82 × 10^12^/L (mean corpuscular volume 73.8 fL, hematocrit 50.3%, and hemoglobin 151 g/L), and platelet count was 230 × 10^9^/L. The serum erythropoietin level was low (1.4 mIU/ml). The bone marrow aspiration showed a hypercellularity with 1.6% blasts (Fig. 1A). G-banding analysis revealed 46, t(6;8)(q27;p12) in 15 cells, 46, idem, add(1)(q32) in one cell, and 46, XX in 4 cells out of 20 metaphases analyzed. Fluorescence in situ hybridization (FISH) analysis revealed a split signal of *FGFR1* in 91% of the bone marrow mononuclear cells. A CT scan showed moderate splenomegaly. *JAK2* V617F mutation was not detected. She was diagnosed with 8p11 myeloproliferative syndrome and was followed up for polycythemia vera without any treatment.

**Fig. 2:**
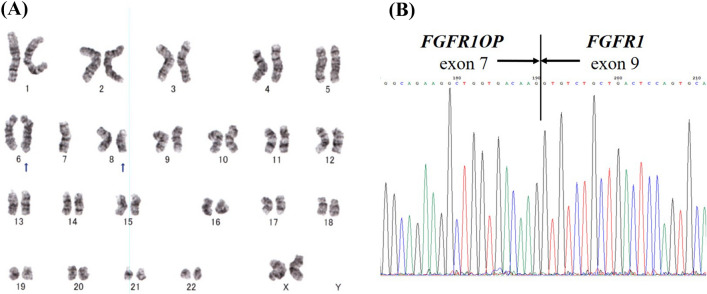
Sequencing and cytogenetic analyses of bone marrow cells at the time of diagnosis of B-ALL. **A** G-banding analysis: the arrows indicate t(6;8)(q27;p11.2). **B** Sequencing analysis of the reverse transcription-polymerase chain reaction product. The vertical line indicates the junction of *FGFR1* and *FGFR1OP*

Four years after the diagnosis, her white blood cell count increased to 161 × 10^9^/L with 2% blasts, and her hemoglobin level and platelet count dropped to 47 g/L and 75 × 10^9^/L, respectively. The bone marrow was hypercellular with 17.8% peroxidase-negative blasts. Flow cytometric analysis showed that the blasts were positive for CD10, CD19, CD22, CD34, and terminal deoxynucleotidyl transferase (TdT) and partially positive for CD13. In addition to t(6;8)(q27;p12), monosomy 7 was detected. Ruxolitinib treatment resulted in no change in the white blood cell count. However, the white blood cell count dropped transiently following the administration of hydroxyurea. Two months later, the blasts in the peripheral blood had increased to 24%. The bone marrow aspiration showed a hypercellular marrow with 70.4% blasts, which expressed the same surface antigens as that of two months before in addition to CD13 (Fig. 1B). Blasts were myeloperoxidase (MPO)-negative by flow cytometric analysis. G-banding analysis revealed 45, XX, t(6;8)(q27;p11.2), -7 in all 20 of the metaphases analyzed (Fig. 2A). Nested reverse transcription-polymerase chain reaction (RT‒PCR) analysis detected the *FGFR1OP-FGFR1* fusion transcript using the *FGFR1OP* and *FGFR1* primers as previously described [, 5, 6]. Sequencing of the PCR product indicated that exon 7 of *FGFR1OP* was fused to exon 9 of *FGFR1* (Fig. 2B). Based on these findings, the patient was diagnosed with B-ALL associated with *FGFR1* rearrangement. She received induction therapy using the L-AdVP regimen with 20 mg/m^2^/day daunorubicin on Days 1–3, 19, 20, 33, and 34, and 1.4 mg/m^2^/day vincristine on Days 1, 8, and 33. L-Asparaginase was administered at 4000 U/day from Day 19 to Day 32. Prednisolone was administered at 40 mg/m^2^/day from Day 1 and was reduced gradually and ceased on Day 27. As the proportion of blasts in the bone marrow was 9.6% after induction therapy, we considered it a failure. She was subsequently treated with a hyper-CVAD regimen at a 20% dose reduction [7], which reduced the percentage of bone marrow blasts (7.8%). Flow cytometric analysis showed that the blasts were positive for CD13, CD34, CD117, and TdT and partially positive for CD19, CD22, CD33, and MPO but negative for CD10 (Fig. 1C). The leukemic phenotype was judged to transform into a mixed phenotype. Two weeks later, blasts in the bone marrow increased to 42%. She was treated with the JALSG ALL-202-O induction regimen but did not achieve complete remission. Then, she received MEC therapy at a 50% dose reduction [8]. Four weeks after MEC therapy, the white blood cell count was 98.1 × 10^9^/L, and the number of blasts in the bone marrow was 40%. After cytoreduction by prednisolone and cytarabine, she started blinatumomab therapy. Eighteen days after the administration of blinatumomab, as the white blood cell count increased to 97.6 × 10^9^/L, hydroxyurea was added. After the first course of blinatumomab, blasts in the bone marrow were 5.8%. The best supportive care was provided due to poor performance status afterward. She died 11 months after the diagnosis of B-ALL.

**Fig. 3:**
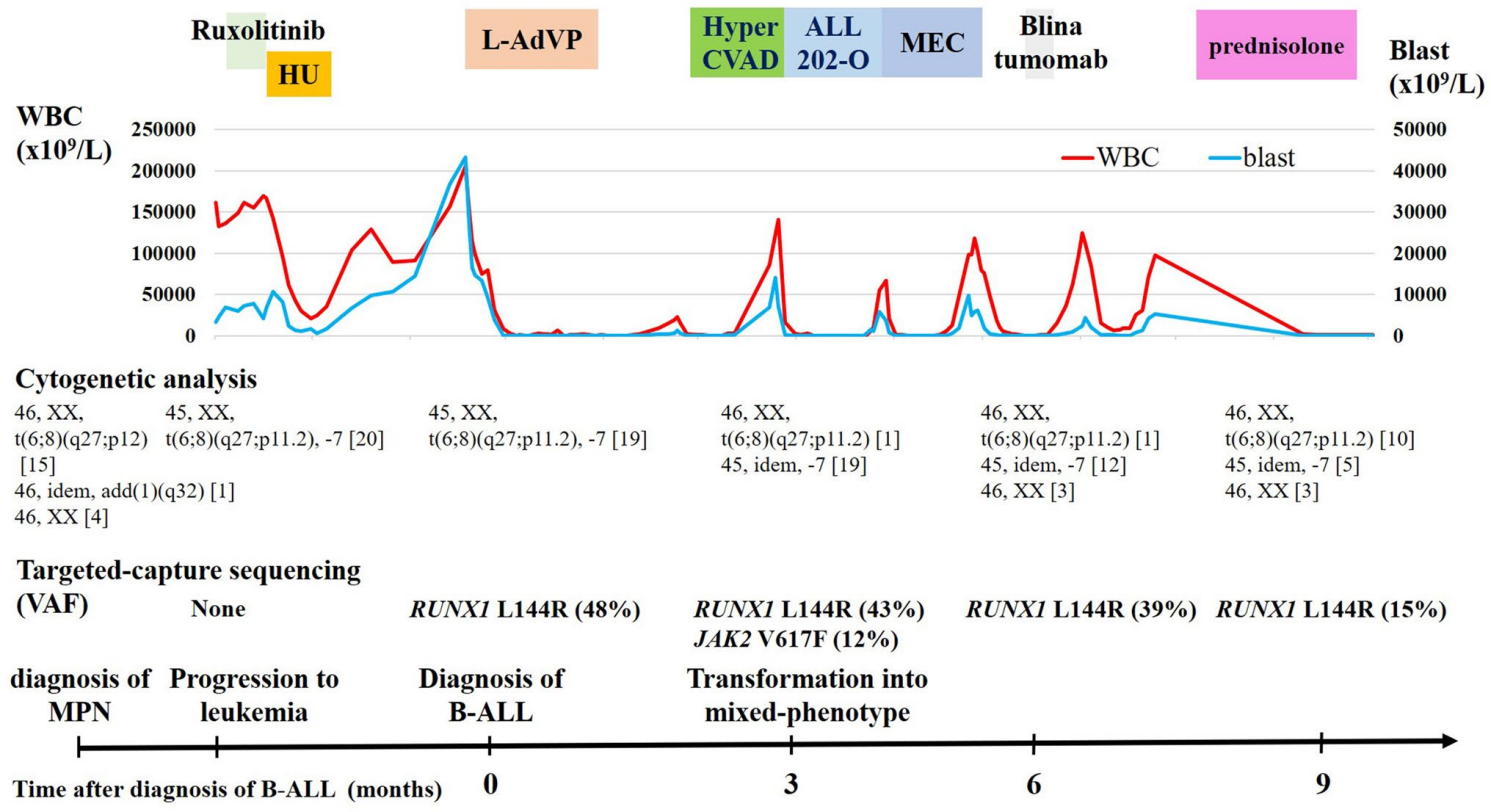
Clinical course and results of cytogenetic and targeted-capture sequencing analyses of bone marrow cells. *MEC* mitoxantrone, etoposide, cytarabine; *VAF* variant allele frequency

We performed targeted-capture sequencing of the patient’s bone marrow cells at the time of the initial increase in blast count, the diagnosis of B-ALL, after the hyper-CVAD regimen, before the administration of blinatumomab and during the administration of prednisolone for a panel of common driver genes implicated in myeloid malignancies [9]. The ALL panel was also used for the sample collected at the time of diagnosis of B-ALL. The analysis was approved by the Institutional Review Boards for Clinical Research of Dokkyo Medical University and Kyoto University in accordance with the Declaration of Helsinki. Written informed consent from the patient was obtained. The clinical course of the patient is summarized in Fig. 3. Although no known driver mutations were identified at the initial increase in blast count, *RUNX1* L144R mutation was identified at a variant allele frequency (VAF) of 48% at the time of diagnosis with B-ALL. After the hyper-CVAD regimen, the *JAK2* V617F mutation emerged at a VAF of 12% in addition to the *RUNX1* L144R mutation at a VAF of 43%. Before the administration of blinatumomab, while the *RUNX1* L144R mutation existed at a VAF of 39%, the *JAK2* mutation disappeared.

## Discussion

This patient was initially diagnosed with MPN with t(6;8)(q27;p12) and was followed up for polycythemia vera. Four years later, she developed acute B-lymphoblastic leukemia with an additional chromosomal abnormality of -7. After two induction regimens, she failed to achieve complete remission, and leukemia transformed into mixed-phenotype leukemia.

EMS was categorized as myeloid/lymphoid neoplasms with *FGFR1* rearrangement in the 2017 revision of the World Health Organization classification of myeloid neoplasms and acute leukemia [10]. To date, 13 translocation partners have been reported [1]. The most common translocation is t(8;13)(p11.2;q12), which generates the *ZMYM2-FGFR1* fusion gene [2]. Patients show eosinophilia and often develop lymphadenopathy and T-lymphoblastic lymphoma. The t(8;22)(p11;q11) generating *BCR-FGFR1* is another common translocation in patients with EMS. Patients with t(8;22)(p11;q11) present with basophilia rather than eosinophilia, which is similar to chronic myeloid leukemia. Most patients with *BCR-FGFR1* develop B-ALL or lymphoma [11]. The prognosis of patients with EMS is poor. The 1-year overall survival is reported to be 43% [12]. Hematopoietic stem cell transplantation is the only curative treatment. The t(6;8)(q27;p11.2)-generating *FGFR1OP-FGFR1* is a rare type of EMS. Of note, patients with t(6;8)(q27;p11.2) often present with polycythemia vera, as seen in this case [5, 6]. In our case, the *FGFR1OP-FGFR1* fusion transcript was generated between exon 9 of *FGFR1* and exon 7 of *FGFR1OP*, as previously reported [6]. Exon 5 or 9 of *FGFR1OP* can also fuse with *FGFR1* [6, 13]. To date, 12 cases with t(6;8)(q27;p11.2) have been reported, and six patients transformed to acute myeloid leukemia [5, 13–15] and one patient developed B-ALL [6] (Table 1). Our case transformed to B-ALL associated with monosomy 7. Notably, the case that Vizmanos reported also carried monosomy 7 at the development of B-ALL [6]. Finally, leukemic cells in our case obtained myeloid markers including MPO in addition to the lymphoid markers after a few courses of chemotherapy for ALL.

**Table 1:** Reported cases with t(6;8)(q27;p11)

Author	Age, sex	Initial diagnosis	Karyotype	Treatment	Transformation	Time to acute leukemia (months)	Outcome
Our case	67, F	PV	46, XX, t(6;8)(q27;p12)	Ruxolitinib, HU	B-ALL	49	Death
Vannier [14]	13, F	CML T-cell lymphoma	46, XX, t(6;8)(q27;p12)	HU, Chemo	Acute leukemia	12	Death
Chaffanet [15] case 1	20, M	MPD	46, XY, t(6;8)(q27;p11)	NA	AML	NA	NA
Chaffanet [15] case 2	27, M	MPD	46, XY, t(6;8)(q27;p11)	NA	AML M1	NA	NA
Popovici [5] case 1	27, M	PV	46, XY, t(6;8)(q27;p11)	HU	AML	60	Death
Popovici [5] case 2	19, M	PV	46, XY, t(6;8)(q27;p11)	Chemo	AML	12	Death
Sohal [13] case 1	M	Ph-negative CML with eosinophilia	46, XY, t(6;8)(q27;p11.2)	BMT	AML	NA	NA
Sohal [13] case 2	F	AML/EMS	46, XX, t(6;8)(q27;p11), + 8, + 10, − 18, − 19, + dic(18;19)(p11.2;13.3)	Chemo	NA	NA	NA
Vizmanos [6] case 1	41, F	PV	46, XX, t(6;8)(q27;p12)	Interferon-a, HU	Ph-negative CML	NA	Alive as of publication
Vizmanos [6] case 2	66, M	B-ALL	45, XY, t(6;8)(q27;p12), − 7	Chemo	NA	NA	Death
Lourenco [16]	12, F	Chronic myeloid neoplasm mimicking PV	46, XX, t(6;8)(q27;p11)	HU	AML	48	Death
Strati [17] case 1	F	Chronic eosinophilic leukemia	46, XX, t(6;8)(q27;p11.23)	HU, HSCT	NA	NA	Death
Strati [17] case 2	F	MPN	46, XX, t(6;8)(q27;p11.23)	HU, Anagrelide	NA	NA	Alive as of publication

*AML* acute myeloid leukemia, *BMT* bone marrow transplantation, *Chemo* chemotherapy, *CML* chronic myeloid leukemia, *HSCT* hematopoietic stem cell transplantation, *HU* hydroxyurea, *MPD* myeloproliferative disorders, *NA* not available, *Ph* Philadelphia chromosome, *PV* polycythemia vera

In our case, the accumulation of chromosomal abnormalities and genetic mutations was observed during the progression of the disease. At the initial increase in blast count, monosomy 7 was found in all of the leukemic cells and no other gene mutations were observed. After the administration of ruxolitinib and hydroxyurea, she was diagnosed with B-ALL and leukemic cells with *RUNX1* mutation expanded at the development of B-ALL. This expansion is probably due to clonal selection associated with those treatments. *RUNX1* mutations were frequently observed in patients with EMS, and all patients with *RUNX1* mutation presented with acute leukemia [17]. Therefore, *RUNX1* mutation may play an important role in the transformation of EMS to leukemia. In our case, the *JAK2* V617F mutation emerged after the hyper-CVAD regimen. At the same time, blasts expressed MPO as well as myeloid surface markers. Considering that the *JAK2* V617F mutation is a characteristic gene mutation of myeloproliferative neoplasms [18–21], the emergence of the *JAK2* V617F mutation might be associated with the appearance of a myeloid phenotype.

As with chronic myeloid leukemia (CML), constitutive activation of tyrosine kinase in hematopoietic stem cells leads to the development of MPN in EMS [2], and the accumulation of gene mutations causes a blast crisis. For CML, tyrosine kinase inhibitors are effective [22–24]. A deep molecular response can be achieved, and progression to blast crisis is rare. For EMS, ponatinib in combination with chemotherapy was reported to be effective for a patient with t(8;22)(p11;q11) as a bridge to hematopoietic stem cell transplantation [4]. In addition, pemigatinib, which is a selective *FGFR1-3* inhibitor, is reported to be a promising drug. The phase 2 study of pemigatinib showed that 75% of patients with EMS achieved complete cytogenetic response [25]. Currently, the prognosis of patients with EMS who progress to leukemia is poor, and hematopoietic stem cell transplantation should be performed for transplant-eligible patients as soon as possible. Information on the accumulation of genetic mutation is helpful in selecting an optimal transplant timing. We expect that next-generation sequencing-based multigene panel testing in hematological malignancies will be introduced to clinical practice shortly.

## Data Availability

The data of this study are available on request from the corresponding author. The data are not publicly available due to privacy.
